# Sibling count and family employment status shape injury patterns and emergency department resource use in pediatric trauma

**DOI:** 10.1186/s12873-026-01589-6

**Published:** 2026-04-20

**Authors:** Mahmut Şahin, Osman Taş, Mehmet Şirin Büyükkaya, Mehmet Yorgun

**Affiliations:** Department of Emergency Medicine, Van Training and Research Hospital, University of Health Sciences, Van, TR65040 Turkey

**Keywords:** Paediatric trauma, Sibling count, Family structure, Emergency department, Resource utilization

## Abstract

**Background:**

Paediatric trauma presentations to emergency departments (EDs) are typically low in severity, yet they constitute a substantial component of ED workload. Although social determinants of health influence paediatric injury risk, the role of family structure in shaping injury patterns and healthcare utilisation remains insufficiently explored.

**Objective:**

To examine associations between sibling count, family characteristics, injury patterns, and ED resource utilisation in paediatric trauma.

**Methods:**

This prospective single-centre observational study included children aged 0–15 years presenting with traumatic injuries between October 2025 and January 2026. Demographic variables, household characteristics (sibling count, parental employment status, caregiver availability), and trauma data were recorded prospectively. Associations between sibling count, injury patterns, and ED resource use were analysed using non-parametric tests and multivariable logistic regression adjusted for age and sex.

**Results:**

Among 408 patients (median age 8 years; 62.3% male), overall injury severity was low (median ISS 2) and not associated with sibling count. Higher sibling counts were independently associated with extremity fractures or dislocations (adjusted OR 1.32 per additional sibling, 95% CI 1.10–1.57), and children with ≥ 3 siblings had higher odds of extremity fractures (adjusted OR 2.76). Head injuries were more frequent among children with fewer siblings. Higher sibling counts were also associated with increased specialist consultation requirements.

**Conclusions:**

Sibling count was associated with injury patterns and selected indicators of ED resource utilisation but not with overall trauma severity. Household context may therefore influence exposure patterns and care pathways in low-acuity paediatric trauma.

**Supplementary Information:**

The online version contains supplementary material available at 10.1186/s12873-026-01589-6.

## Introduction

Paediatric trauma is a frequent cause of emergency department (ED) utilisation and a significant source of preventable morbidity in children. A substantial body of epidemiological research consistently demonstrates that unintentional injuries account for the preponderance of paediatric trauma presentations, with the majority of these injuries being of low severity. Extremity injuries are a prevalent occurrence among paediatric patients presenting to emergency care services [1–3]. Previous studies have shown that paediatric injuries frequently occur in home environments and are commonly related to falls [4, 5]. While these clinical patterns are well documented, increasing attention has focused on broader contextual factors that may influence paediatric injury epidemiology.

Research on social determinants of health has highlighted the importance of household and socioeconomic environments in shaping injury risk and healthcare utilisation in children. Factors such as parental employment, supervision patterns, and household structure have been demonstrated to influence both exposure to injury and decisions to seek medical care [6–8]. However, the majority of paediatric trauma studies have focused primarily on injury mechanisms or physiological severity, rather than examining how household context may influence injury patterns and ED resource use.

Household structure represents one potential contextual factor affecting paediatric injury exposure. The number of siblings may be indicative of aspects of the family environment, such as the quality of supervision, the dynamics of peer interactions, and the household crowding. It is important to note that the number of siblings should not be interpreted as a direct causal determinant of injury risk. Rather, it should be considered a contextual indicator embedded within broader social and household environments.

From an ED perspective, contextual factors may also help explain variation in ED resource utilisation. The management of paediatric trauma frequently encompasses imaging, consultation, and observation decisions that are not exclusively determined by the severity of the injury [9–15]. It has been demonstrated by preceding studies that low-acuity paediatric emergency department (ED) visits have the capacity to engender significant demand for diagnostic testing and consultation, notwithstanding favourable clinical outcomes [16, 17].

The objective of this observational study was to examine the association between the number of siblings a patient has had, family characteristics, injury patterns, and selected indicators of the utilisation of emergency department resources among paediatric trauma patients presenting to the emergency department. The hypothesis was formulated that the number of siblings would be associated with differences in the distribution of injuries, particularly those affecting the extremities, but not with the overall severity of trauma.

## Methods

### Study design and setting

This prospective single-centre observational study was conducted in the emergency department of Van Training and Research Hospital, a tertiary-care academic centre with a high-volume paediatric emergency service. Consecutive paediatric trauma patients presenting between 1 October 2025 and 1 January 2026 were evaluated.

### Study population

Children aged 0–15 years who presented with traumatic injuries were eligible for inclusion. Patients were included in the study if informed consent had been obtained from a legal guardian and if complete clinical and sociodemographic data were available, including sibling count, parental employment status, and caregiver availability. Patients presenting with non-traumatic conditions, incomplete trauma records, or missing consent were excluded from the study.

### Data collection

The data were prospectively recorded using structured case report forms. The collected variables encompassed demographic characteristics (age and sex), household characteristics (sibling count, caregiver availability, maternal employment status, and paternal employment status), and trauma-related variables. The term “sibling count” is defined as the number of children residing in the same household, with the exception of the index patient. The term ‘caregiver availability’ was defined as the presence of a non-parental caregiver who was responsible for supervising the child at the time of injury.

The trauma-related variables encompassed the following: the trauma mechanism, the incident location, the injured anatomical region, and the Injury Severity Score (ISS). The operational variables encompassed within the emergency department included the utilisation of diagnostic imaging procedures, laboratory testing, consultations with specialist medical practitioners, procedural interventions, the duration of patient stay in the emergency department, hospital admission, and the patient’s discharge status.

### Study outcomes

The primary objective of the study was to evaluate the association between the number of siblings and indicators of emergency department resource utilisation. Subsequent analyses investigated the correlation between the number of siblings and injury characteristics, encompassing injury patterns, trauma mechanisms, and injury locations.

For regression analyses, extremity fracture or dislocation was selected as the primary injury outcome because it represented the most common clinically significant structural injury observed within the cohort.

### Sample size considerations

The temporal framework of the study and the strategy for patient inclusion were determined on the basis of the anticipated volume of paediatric trauma presentations at the study centre. It was determined that a target sample size of approximately 400 patients would be adequate to ensure a sufficient number of outcome events for multivariable modelling. Preliminary epidemiological data and preliminary institutional observations suggested that extremity fractures and dislocations would represent approximately 10–15% of paediatric trauma presentations. Given the anticipated event frequency, it was expected that a cohort of approximately 400 patients would provide an adequate events-per-variable ratio for logistic regression analyses while maintaining stable model estimation.

### Statistical analysis

Continuous variables were summarised as medians with interquartile ranges (IQR), while categorical variables were reported as frequencies and percentages. Group comparisons were performed using the Mann–Whitney U test for continuous variables and the chi-square test for categorical variables. In instances where comparisons encompassed more than two groups, the Kruskal–Wallis test was employed. The effect sizes were reported alongside p-values using rank biserial correlations for Mann–Whitney U tests and Cramér’s V for chi-square tests.

The sibling count was analysed both as a continuous variable and as a categorical variable (with a minimum of three siblings). The continuous approach was utilised to preserve potential dose–response relationships, while the categorical threshold was employed to represent relatively larger household structures within the study population.

Multivariable logistic regression analyses were performed to identify factors independently associated with selected outcomes, including extremity fracture or dislocation and head injury. Variables that demonstrated statistical significance in univariate analyses were considered for inclusion in multivariable models. Furthermore, age and sex were incorporated into the multivariable models as clinically relevant adjustment variables, irrespective of statistical significance.

Multicollinearity among predictors was assessed using variance inflation factors (VIF). Variables demonstrating strong collinearity were excluded from the final model in order to ensure model stability. The performance of the model was evaluated using discrimination and model-fit indices, including the area under the receiver operating characteristic curve (AUC) and the Akaike Information Criterion (AIC). Statistical significance was defined as *p* < 0.05.

The presence of missing data was closely monitored throughout the study period, and complete-case analysis was performed when the proportion of missing data was minimal.

## Results

The study comprised a total of 408 paediatric trauma patients. The median age of the subjects was 8 years (interquartile range [ IQR]: 4–11), and 62.3% of the patients were male. The median sibling count was 2 (interquartile range 2–3). The majority of injuries (47.5%) occurred in the home environment, while a significant proportion (35.0%) occurred in outdoor settings. The most prevalent trauma mechanism was identified as falls, accounting for 74.0% of cases. The overall severity of injury was low, with a median Injury Severity Score (ISS) of 2 (interquartile range [IQR] 1–4). The baseline demographic, household and trauma characteristics of the cohort are summarised in Table 1.

**Table 1:** Baseline characteristics of the study population (*n* = 408). Baseline demographic, family-related, trauma-related, and emergency department operational characteristics of pediatric patients included in the study. Continuous variables are presented as median (IQR), and categorical variables as number (%). Injury severity was assessed using the Injury Severity Score (ISS). Caregiver refers to a non-parental caregiver

Characteristic	Value
Demographic and family characteristics	
Age, years	8.0 (4.0–11.0)
Male sex	254 (62.3%)
Sibling count	2.0 (2.0–3.0)
Caregiver	23 (5.6%)
Maternal age, years	35.0 (30.0–40.0)
Paternal age, years	39.0 (35.0–44.3)
Family age difference, years	4.0 (2.0–6.0)
Family age mean, years	37.0 (32.5–41.5)
Trauma-related characteristics	
Injury Severity Score (ISS)	2.0 (1.0–4.0)
ED length of stay, hours	1.0 (0.5–2.0)
Hospital invoice	249.0 (129.0–523.5)
Incident location	
Home	194 (47.5%)
School	71 (17.4%)
Outdoors	143 (35.0%)
Trauma mechanism	
Fall	302 (74.0%)
Impact	103 (25.2%)
Motor vehicle accident	2 (0.5%)
Bicycle injury	1 (0.2%)
Injury patterns	
Head injury	143 (35.0%)
Neck injury	9 (2.2%)
Chest injury	14 (3.4%)
Abdominal injury	5 (1.2%)
Knee injury	23 (5.6%)
Hip injury	14 (3.4%)
Shoulder injury	14 (3.4%)
Elbow injury	23 (5.6%)
Forearm injury	17 (4.2%)
Hand / wrist injury	94 (23.0%)
Foot injury	69 (16.9%)
Any extremity injury	215 (52.7%)
Upper extremity injury	146 (35.8%)
Lower extremity injury	84 (20.6%)
Upper and lower extremity injury	15 (3.7%)
Multiple trauma	29 (7.1%)

Continuous variables are presented as median (interquartile range, 25th–75th percentiles). Categorical variables are presented as number (%)

Soft tissue injuries constituted the majority of diagnoses (82.8%), followed by upper extremity fractures (11.0%). Other diagnoses, including but not limited to lower extremity fractures, nursemaid’s elbow, and cranial fractures with intracranial haemorrhage, were observed less frequently. The distribution of trauma-related diagnoses is presented in Table 2.

**Table 2:** Distribution of trauma-related diagnoses. Distribution of trauma-related diagnoses among pediatric patients presenting to the emergency department. Diagnoses are reported as number and percentage of the total study population (*n* = 408)

Diagnosis category	*n* (%)
Soft tissue injury	338 (82.8%)
Upper extremity fracture	45 (11.0%)
Lower extremity fracture	6 (1.5%)
Nursemaid’s elbow	7 (1.7%)
Nasal fracture	3 (0.7%)
Cranial fracture with intracranial hemorrhage	6 (1.5%)
Vertebral fracture	1 (0.2%)
Foreign body (soft tissue / nasal)	2 (0.5%)

The sibling count exhibited multiple associations with injury patterns. Children with extremity injuries exhibited higher median sibling counts in comparison with those not exhibiting extremity involvement (3 [interquartile range (IQR): 2–4] vs. 2 [IQR: 1–3]; *p* = 0.003). Analogous patterns were observed for upper extremity injuries (*p* = 0.019) and hand or wrist injuries (*p* = 0.016). Furthermore, children with upper extremity fractures exhibited higher sibling counts, with a mean of 3 (interquartile range: 2–4) compared to 2 (interquartile range: 1.5–3.0); *p* = 0.005. When extremity fractures and dislocations were analysed as a composite outcome, sibling count remained significantly higher among affected patients (*p* = 0.005). The detailed associations between sibling count and trauma-related variables are presented in Table 3.

**Table 3:** Association between sibling count and trauma-related variables Association between sibling count and selected demographic variables, injury characteristics, household factors, and emergency department resource utilization indicators. Sibling count is presented as median (IQR). Group comparisons were performed using the Mann–Whitney U test for two-group analyses and the Kruskal–Wallis test for comparisons involving more than two groups. Caregiver refers to a non-parental caregiver

Variable	Categories	Sibling count, median (IQR)	*p* value	Effect size
Sex	Female vs. Male	2 (2–3) vs. 2 (1–3)	0.644	0.03
ED arrival mode	Walk-in vs. EMS	2 (2–4) vs. 2 (1–3)	0.515	0.07
Incident location	Home / School / Outdoors	2 (1–3) / 3 (2–4) / 2 (2–4)	0.032	0.02
Head injury	Absent vs. Present	3 (2–4) vs. 2 (1–3)	< 0.001	0.23
Neck injury	Absent vs. Present	2 (2–3) vs. 3 (2–3)	0.429	0.15
Chest injury	Absent vs. Present	2 (2–3) vs. 3 (1–3)	0.860	0.03
Abdominal injury	Absent vs. Present	2 (2–3) vs. 3 (2–3)	0.441	0.19
Hip injury	Absent vs. Present	2 (2–3.8) vs. 2.5 (2–3)	0.878	0.02
Shoulder injury	Absent vs. Present	2 (2–3.8) vs. 2.5 (2–3)	0.878	0.02
Elbow injury	Absent vs. Present	2 (2–3) vs. 2 (1–3)	0.678	0.05
Forearm injury	Absent vs. Present	2 (2–3) vs. 3 (1–5)	0.232	0.17
Foot injury	Absent vs. Present	2 (1–3) vs. 3 (2–3)	0.350	0.07
Hand/wrist injury	Absent vs. Present	2 (1–3) vs. 3 (2–4)	0.016	0.16
Any extremity injury	Absent vs. Present	2 (1–3) vs. 3 (2–4)	0.003	0.17
Upper extremity injury	Absent vs. Present	2 (1.2–3.0) vs. 3 (2–4)	0.019	0.14
Lower extremity injury	Absent vs. Present	2 (1–4) vs. 2.5 (2–3)	0.453	0.05
Multiple trauma	Absent vs. Present	2 (2–3.5) vs. 3 (2–3)	0.935	0.01
Maternal employment	Unemployed vs. Employed	2 (2–4) vs. 2 (2–3)	0.135	0.13
Paternal employment	Unemployed vs. Employed	3 (2–4) vs. 2 (2–3)	0.034	0.26
Family employment	Unemployed vs. Employed	3 (2–4) vs. 2 (2–3)	0.026	0.28
Caregiver status	None vs. Has caregiver	2 (2–4) vs. 2 (1–2.5)	0.008	0.32
Upper extremity fracture/dislocation	Absent vs. Present	2 (2–3) vs. 3 (2–4)	0.014	0.21
Lower extremity fracture	Absent vs. Present	2 (2–3) vs. 4.5 (2.2–6.0)	0.162	0.33
Upper extremity fracture	Absent vs. Present	2 (1.5–3.0) vs. 3 (2–4)	0.005	0.25
Any extremity fracture/dislocation	Absent vs. Present	2 (1.5–3.0) vs. 3 (2–4)	0.005	0.23
Cranial fracture / intracranial hemorrhage	Absent vs. Present	2 (2–3) vs. 3 (0.8–3.8)	0.824	0.05
Consultation need	No vs. Yes	2 (1–3) vs. 3 (2–4)	0.007	0.24
ED admission	No vs. Yes	2 (2–3) vs. 3 (2.2–3.8)	0.369	0.16

Values are presented as median (interquartile range). Mann–Whitney U test was used for two-group comparisons and the Kruskal–Wallis test for comparisons involving more than two groups. Caregiver was defined as a non-family caregiver. Effect sizes were reported as rank biserial correlations (Mann–Whitney U), and epsilon-squared (ε²) for Kruskal–Wallis tests

A statistically significant association was identified between head injuries and the number of siblings in the subject group. Children presenting with head injury had lower sibling counts compared with those without head injury (median 2 [IQR 1–3] vs. 3 [IQR 2–4]; *p* < 0.001). Supplementary analyses further demonstrated that children presenting with head injuries were younger and more frequently transported by emergency medical services (Supplementary Table S1).

Extremity fracture or dislocation occurred in 58 patients (14.2%). Patients with this outcome had a higher median sibling count compared with those without fracture or dislocation (3 [ IQR 2–4] vs. 2 [ IQR 2–3]; *p* = 0.005). When the analysis was conducted categorically, children with three or more siblings were more frequently represented in the fracture or dislocation group (67.2% vs. 43.1%; *p* = 0.001). A detailed comparison between these groups is presented in Table 4.

**Table 4:** Comparison of patients with and without extremity fracture or dislocation Comparison of demographic characteristics, injury mechanisms, injury locations, and family-related variables between pediatric trauma patients with and without extremity fracture or dislocation. Continuous variables are presented as median (IQR), and categorical variables as number (%). Group comparisons were performed using the Mann–Whitney U test for continuous variables and chi-square or Fisher’s exact test for categorical variables. Effect sizes are reported as rank biserial correlation (continuous variables) and Cramér’s V (categorical variables)

Variable	Category	No fracture/dislocation (*n* = 350)	Fracture/dislocation (*n* = 58)	Total	*p* value	Effect size
Age	Median (IQR)	8.0 (4.0–11.0)	9.0 (3.0–12.0)	8.0 (4.0–11.0)	0.447	0.06
Sex	Female	132 (37.7)	22 (37.9)	154 (37.7)	1.000	< 0.01
Sex	Male	218 (62.3)	36 (62.1)	254 (62.3)		
ED arrival	Walk-in	318 (90.9)	55 (94.8)	373 (91.4)	0.455	0.05
ED arrival	EMS	32 (9.1)	3 (5.2)	35 (8.6)		
Incident location	Home	169 (48.3)	25 (43.1)	194 (47.5)	0.765	0.04
Incident location	School	60 (17.1)	11 (19.0)	71 (17.4)		
Incident location	Outdoors	121 (34.6)	22 (37.9)	143 (35.0)		
Trauma mechanism	Fall	256 (73.1)	46 (79.3)	302 (74.0)	0.727	0.06
Trauma mechanism	Impact	91 (26.0)	12 (20.7)	103 (25.2)		
Trauma mechanism	Motor vehicle accident	2 (0.6)	0 (0.0)	2 (0.5)		
Trauma mechanism	Bicycle injury	1 (0.3)	0 (0.0)	1 (0.2)		
Sibling count	Median (IQR)	2.0 (2.0–3.0)	3.0 (2.0–4.0)	2.0 (2.0–3.0)	0.005	0.23
Sibling ≥ 3	No	199 (56.9)	19 (32.8)	218 (53.4)	0.001	0.17
Sibling ≥ 3	Yes	151 (43.1)	39 (67.2)	190 (46.6)		
Maternal age	Median (IQR)	35.0 (30.0–40.0)	37.0 (32.2–40.0)	35.0 (30.0–40.0)	0.086	0.14
Paternal age	Median (IQR)	39.0 (35.0–44.0)	40.5 (36.2–45.0)	39.0 (35.0–44.2)	0.102	0.13
Maternal employment	Unemployed	307 (87.7)	51 (87.9)	358 (87.7)	1.000	< 0.01
Maternal employment	Employed	43 (12.3)	7 (12.1)	50 (12.3)		
Paternal employment	Unemployed	18 (5.1)	5 (8.6)	23 (5.6)	0.449	0.05
Paternal employment	Employed	332 (94.9)	53 (91.4)	385 (94.4)		
Family employment	Unemployed	16 (4.6)	5 (8.6)	21 (5.1)	0.331	0.06
Family employment	Employed	334 (95.4)	53 (91.4)	387 (94.9)		
Caregiver	No caregiver	330 (94.3)	55 (94.8)	385 (94.4)	1.000	0.01
Caregiver	Has caregiver	20 (5.7)	3 (5.2)	23 (5.6)		

Values are presented as median (interquartile range) or number (%)

In multivariable logistic regression analysis, adjusting for age and sex, the sibling count remained independently associated with extremity fracture or dislocation. The presence of each additional sibling was associated with an increased risk of extremity fracture or dislocation (adjusted OR 1.32, 95% CI 1.10–1.57; *p* = 0.002). In a similar vein, the presence of three or more siblings was found to be associated with an elevated risk of extremity fracture or dislocation in comparison with siblings of a smaller number (adjusted OR 2.76, 95% CI 1.50–5.07; *p* = 0.001). The complete regression model results are presented in Table 5.

**Table 5:** Multivariable logistic regression analysis of factors associated with extremity fracture or dislocation Results of univariable and multivariable logistic regression analyses evaluating factors associated with extremity fracture or dislocation among pediatric trauma patients. Odds ratios (ORs) with 95% confidence intervals (CIs) are reported. The multivariable model included clinically relevant variables identified in univariable analyses, with adjustment for age and sex. Variables demonstrating significant multicollinearity were excluded from the final model. Model performance was evaluated using discrimination (AUC) and model fit indices (AIC)

Variable	Univariate OR (95% CI)	*P* value	Adjusted OR (95% CI)	*P* value
Male sex	0.99 (0.56–1.76)	0.975	1.04 (0.58–1.87)	0.897
Paternal employment (employed vs. unemployed)	0.54 (0.20–1.61)	0.293	—	—
Family employment ≥ 1	0.51 (0.18–1.44)	0.204	—	—
Age (child)	1.02 (0.96–1.09)	0.522	0.99 (0.92–1.06)	0.752
Maternal age	1.03 (0.99–1.08)	0.153	—	—
Paternal age	1.03 (0.99–1.07)	0.157	—	—
Sibling count (per additional sibling)	1.31 (1.10–1.54)	0.002	1.32 (1.10–1.57)	0.002
Sibling count ≥ 3	2.71 (1.50–4.87)	< 0.001	2.76 (1.50–5.07)	0.001

OR: Odds ratio; CI: Confidence interval; VIF: Variance inflation factor. Adjusted odds ratios were calculated using a multivariable logistic regression model

Logistic regression model specification: logit(P(extremity fracture/dislocation)) = β0 + β1(sex) + β2(child age) + β3(sibling count) + β4(sibling count ≥ 3)

Model selection: Maternal age, paternal age, and family employment status were excluded from the multivariable model due to significant multicollinearity (VIF ≈ 939 and *r* > 0.85). Child age was retained as the primary age‑related predictor

Note: Sibling count was included in the multivariable model as a continuous variable (per additional sibling). For interpretability, an additional categorical analysis using a threshold of ≥ 3 siblings was also performed

Model performance: The multivariable logistic regression model was statistically significant (χ²[3] = 9.74, *p* = 0.021). Model fit indices indicated limited explanatory capacity (McFadden R² = 0.03; Cox–Snell R² = 0.02; Tjur R² = 0.03). The Akaike Information Criterion (AIC) was 331.89. The model demonstrated modest discrimination (AUC = 0.61). Multicollinearity diagnostics showed no evidence of collinearity among predictors (all VIF values < 1.13)

Further multivariable analyses were conducted for head injury. The findings of the study demonstrated that a decrease in age was independently associated with elevated odds of head injury (adjusted OR 0.86 per year increase, 95% CI 0.81–0.91; *p* < 0.001). Conversely, the presence of three or more siblings was associated with diminished odds of head injury (adjusted OR 0.60, 95% CI 0.38–0.94; *p* = 0.027). Detailed results are displayed in the supplementary table (Supplementary Table S2).

Children requiring specialist consultation had higher sibling counts than those managed without consultation (median 3 [interquartile range (IQR) 2–4] vs. 2 [IQR 1–3]; *p* = 0.007). Supplementary analyses exploring factors associated with consultation requirement are presented in supplementary tables S3-S4.

## Discussion

In this prospective observational study of paediatric trauma patients presenting to the emergency department, the number of siblings was not associated with overall injury severity. However, the study found that these associations were contingent on differences in injury patterns and selected indicators of emergency department resource utilisation. These findings emerged within a cohort predominantly characterised by low injury severity, which is consistent with large epidemiological series showing that most paediatric trauma presentations involve low-acuity injuries rather than severe trauma [1–3].

The absence of an association between the number of siblings and overall trauma severity is consistent with previous paediatric trauma literature, which demonstrates that severe outcomes are relatively uncommon in unselected emergency department trauma populations [1–3]. In this context, the observed associations between the number of siblings and the distribution of injuries suggest that household characteristics may influence the environments in which injuries occur, rather than the physiological severity of the injuries themselves. This interpretation is consistent with a growing body of literature highlighting the role of social and household determinants in shaping paediatric injury risk and healthcare utilisation [6–8].

A salient finding of the present study was the association between a greater number of siblings and an increased frequency of extremity injuries, particularly upper extremity and hand–wrist injuries, including fractures. Extremity injuries represent a substantial proportion of paediatric emergency department trauma encounters and frequently require imaging and orthopaedic consultation even when overall injury severity is low [9–12]. In the present analysis, children with extremity fractures or dislocations exhibited higher levels of sibling counts, and this association remained statistically significant in multivariable logistic regression models. Each additional sibling was found to be associated with increased odds of extremity fracture or dislocation, and children with three or more siblings had approximately twofold higher odds of sustaining such injuries. Despite the regression model’s modest explanatory capacity, the findings indicate that household size may be associated with variations in everyday injury exposure environments.

In contrast, a higher prevalence of head injuries was observed among children with fewer siblings. Further analyses indicated that younger age was strongly associated with head injury presentations, while higher sibling counts were associated with lower odds of head injury. Of particular significance was the finding that the number of siblings was not associated with markers of severe head trauma, including cranial fracture or intracranial haemorrhage. These findings are consistent with contemporary paediatric head trauma literature, which demonstrates that the majority of emergency department presentations involve mild injury mechanisms and low rates of clinically significant intracranial injury [13, 18, 19]. Taken together, these observations suggest that the number of siblings a person has had may be associated with variation in injury patterns rather than with the risk of severe traumatic outcomes.

In the present study, sibling count was analysed both as a continuous variable and as a categorical variable using a threshold of three siblings. The threshold in question was selected to represent relatively larger household environments within the study population. In the eastern regions of Türkiye, where the study centre is located, household sizes are typically larger than the national average. According to data from the Turkish Statistical Institute (TÜİK), the average household size in these regions exceeds four individuals [20]. In this context, the presence of three or more siblings may be indicative of households characterised by increased population density and shared living arrangements. Such environments may plausibly influence patterns of play, supervision, and daily activity among children, potentially contributing to differences in injury exposure.

The study also identified associations between the number of siblings and the contextual characteristics of injury. A greater proportion of children who sustained injuries in school settings and in households characterised by parental unemployment or limited caregiver support exhibited higher sibling counts. These findings suggest that the number of siblings in a child’s family environment may serve as a proxy indicator reflecting broader ecological and social environments, including supervision patterns, family structure, and socioeconomic conditions. This interpretation aligns with extant literature on social determinants of health, which demonstrates that family structure, caregiving environments, and socioeconomic vulnerability influence paediatric injury exposure and healthcare utilisation [21–24]. Consequently, the sibling count should be interpreted as a contextual marker rather than as a causal determinant of injury risk.

From the standpoint of the emergency department, these patterns may also help explain variation in the utilisation of resources among low-acuity paediatric trauma presentations. In the present study, an association was identified between higher sibling counts and increased specialist consultation requirements, as well as injury patterns that commonly necessitate diagnostic imaging or orthopaedic evaluation. It has been demonstrated by preceding studies that a significant proportion of emergency department resource utilisation in paediatric trauma originates from low-acuity injuries as opposed to severe trauma [9, 10]. The findings of this study therefore suggest that household characteristics may exert an indirect influence on emergency department workload through their association with common injury patterns that require clinical evaluation.

It is imperative to exercise caution when interpreting these findings. The observational design precludes causal inference, and the observed associations are likely to reflect complex interactions between family structure, supervision environments, behavioural exposure, and socioeconomic context. Rather than being a direct determinant of injury risk, the number of siblings should be considered a contextual variable embedded within broader social and caregiving environments.

From a prevention perspective, the clustering of extremity injuries and school-based injury contexts among children from larger households may highlight opportunities for targeted injury prevention strategies. It is important to note that school environments and recreational activities represent common settings for paediatric extremity injuries. Therefore, prevention programmes in these contexts may be particularly relevant. The incorporation of household context into paediatric injury surveillance may also facilitate the development of more refined prevention strategies, with a focus on everyday injury environments rather than severe trauma mechanisms.

### Clinical and public health implications

It is submitted that a more comprehensive understanding of the contextual factors associated with patterns of paediatric injury may facilitate the development of more targeted prevention strategies. The present findings suggest that household characteristics, including family size, may be associated with predictable injury patterns such as extremity injuries occurring during routine activities. For emergency departments serving populations with larger household sizes, awareness of these patterns may help clinicians to anticipate common diagnostic and consultation pathways associated with low-acuity paediatric trauma presentations without implying increased injury severity.

### Strengths and limitations

This study has several strengths. The prospective design and consecutive patient inclusion reduced selection bias and improved internal validity. In addition, the structured assessment of household characteristics enabled the evaluation of contextual variables that are seldom captured in routine trauma registries. The employment of multivariable modelling also facilitated the exploration of independent associations between household factors and injury outcomes.

It is imperative to acknowledge the limitations of the present study. Firstly, the single-centre design may limit the generalisability of the findings to other healthcare systems and populations. Secondly, a number of contextual factors were not directly measured, including the quality of supervision, the age distribution of siblings, and behavioural dynamics within households. Thirdly, the observational nature of the study precludes the ability to make causal inferences, and residual confounding cannot be excluded. Finally, the cohort consisted predominantly of low-acuity trauma cases, which may limit the applicability of the findings to more severe trauma populations.

## Conclusions

Sibling count was associated with differences in injury patterns and selected indicators of emergency department resource utilisation among paediatric trauma patients. However, no association was observed with overall injury severity. These findings suggest that household context may influence injury exposure and subsequent care pathways in paediatric trauma populations with predominantly low-acuity injuries.

## Electronic supplementary material

Below is the link to the electronic supplementary material.

**Supplementary Material 1**: Supplementary Table S1. Baseline characteristics according to head injury status Comparison of demographic, injury-related, and family characteristics between pediatric trauma patients with and without head injury. Continuous variables are presented as median (IQR), and categorical variables as number (%). Group comparisons were performed using the Mann–Whitney U test and chi-square test. Effect sizes are reported as rank biserial correlation and Cramér’s V. Supplementary Table S2. Multivariable logistic regression analysis of factors associated with head injury Odds ratios (ORs) with 95% confidence intervals (CIs) are presented. Variables included in the multivariable model were selected based on clinical relevance and univariable analyses. Multicollinearity diagnostics were performed, and highly correlated variables were excluded. Model performance was assessed using AUC and AIC. Supplementary Table S3. Baseline characteristics according to specialist consultation requirement in the emergency department (N = 408) Continuous variables are presented as median (IQR), and categorical variables as number (%). Group comparisons were performed using the Mann–Whitney U test and chi-square test. Effect sizes are reported as rank biserial correlation and Cramér’s V. Consultation requirement refers to specialist consultation requested during emergency department evaluation. Supplementary Table S4. Multivariable logistic regression analysis of factors associated with specialist consultation requirement Odds ratios (ORs) with 95% confidence intervals (CIs) are presented. Variables were selected based on clinical relevance and univariable analyses. Multicollinearity was assessed using variance inflation factors (VIF), and highly correlated variables were excluded. Model performance was evaluated using AUC, AIC, and pseudo-R² indices

## Data Availability

The data supporting the findings of this study are available from the corresponding author upon reasonable request.

## Declarations

### Presented at a meeting

No.

## Abbreviations

ED: Emergency Department
ISS: Injury Severity Score
EMS: Emergency Medical Services
IQR: Interquartile Range
OR: Odds Ratio
CI: Confidence Interval
VIF: Variance Inflation Factor
AUC: Area Under the Curve
AIC: Akaike Information Criterion
