# Supplementary material for: Sibling count and family employment status shape injury patterns and emergency department resource use in pediatric trauma
Source: BMC Emerg Med. 2026 Apr 20;26:162. doi: 10.1186/s12873-026-01589-6 (PMC13231668; doi:10.1186/s12873-026-01589-6)
Supplement: Supplementary file 1 — Supplementary Material 1: Supplementary Table S1. Baseline characteristics according to head injury status Comparison of demographic, injury-related, and family characteristics between pediatric trauma patients with and without head injury. Continuous variables are presented as median (IQR), and categorical variables as number (%). Group comparisons were performed using the Mann–Whitney U test and chi-square test. Effect sizes are reported as rank biserial correlation and Cramér’s V. Supplementary Table S2. Multivariable logistic regression analysis of factors associated with head injury Odds ratios (ORs) with 95% confidence intervals (CIs) are presented. Variables included in the multivariable model were selected based on clinical relevance and univariable analyses. Multicollinearity diagnostics were performed, and highly correlated variables were excluded. Model performance was assessed using AUC and AIC. Supplementary Table S3. Baseline characteristics according to specialist consultation requirement in the emergency department (N = 408) Continuous variables are presented as median (IQR), and categorical variables as number (%). Group comparisons were performed using the Mann–Whitney U test and chi-square test. Effect sizes are reported as rank biserial correlation and Cramér’s V. Consultation requirement refers to specialist consultation requested during emergency department evaluation. Supplementary Table S4. Multivariable logistic regression analysis of factors associated with specialist consultation requirement Odds ratios (ORs) with 95% confidence intervals (CIs) are presented. Variables were selected based on clinical relevance and univariable analyses. Multicollinearity was assessed using variance inflation factors (VIF), and highly correlated variables were excluded. Model performance was evaluated using AUC, AIC, and pseudo-R² indices [file 12873_2026_1589_MOESM1_ESM.docx]

# Supplementary Tables

# Supplementary Table S1. Baseline characteristics according to head injury status

| Variable | No head injury (n=265) | Head injury (n=143) | p value | Effect size |
| --- | --- | --- | --- | --- |
| Age (years) | 10 (6–12) | 5 (2–9) | <0.001 | 0.40 |
| Sex |  |  | 0.744 | 0.02 |
| Female | 98 (37.0) | 56 (39.2) |  |  |
| Male | 167 (63.0) | 87 (60.8) |  |  |
| ED arrival with EMS | 13 (4.9) | 22 (15.4) | 0.001 | 0.18 |
| Incident location |  |  | 0.007 | 0.16 |
| Home | 111 (41.9) | 83 (58.0) |  |  |
| School | 53 (20.0) | 18 (12.6) |  |  |
| Outdoors | 101 (38.1) | 42 (29.4) |  |  |
| Trauma mechanism |  |  | 0.333 | 0.09 |
| Fall | 199 (75.1) | 103 (72.0) |  |  |
| Impact | 64 (24.2) | 39 (27.3) |  |  |
| Motor vehicle accident | 2 (0.8) | 0 (0.0) |  |  |
| Bicycle injury | 0 (0.0) | 1 (0.7) |  |  |
| Sibling count | 3 (2–4) | 2 (1–3) | <0.001 | 0.23 |
| Sibling count ≥3 |  |  | <0.001 | 0.19 |
| <3 siblings | 123 (46.4) | 95 (66.4) |  |  |
| ≥3 siblings | 142 (53.6) | 48 (33.6) |  |  |
| Maternal age (years) | 36 (32–41) | 32 (29–36) | <0.001 | 0.38 |
| Paternal age (years) | 41 (36–45) | 36 (32–40) | <0.001 | 0.37 |
| Paternal unemployment | 20 (7.5) | 3 (2.1) | 0.040 | 0.11 |
| Maternal unemployment | 230 (86.8) | 128 (89.5) | 0.522 | 0.04 |
| Family unemployment |  |  | 0.070 | 0.10 |
| Unemployed | 18 (6.8) | 3 (2.1) |  |  |
| Employed | 247 (93.2) | 140 (97.9) |  |  |
| Caregiver present |  |  | 0.801 | 0.02 |
| No caregiver | 249 (94.0) | 136 (95.1) |  |  |
| Has caregiver | 16 (6.0) | 7 (4.9) |  |  |

Effect sizes are presented as rank biserial correlations (Mann–Whitney U) and Cramér’s V (chi-square tests).

# Supplementary Table S2. Multivariable logistic regression analysis of factors associated with head injury

| Variable | Univariate OR (95% CI) | P value | Adjusted OR (95% CI) | P value |
| --- | --- | --- | --- | --- |
| Male sex | 0.91 (0.60–1.39) | 0.665 | 1.03 (0.66–1.62) | 0.897 |
| Paternal employment (employed vs unemployed) | 3.81 (1.11–13.05) | 0.033 | 3.41 (0.94–12.37) | 0.062 |
| Family employment ≥1 | 3.40 (0.98–11.75) | 0.053 | — | — |
| Age (child) | 0.84 (0.80–0.89) | <0.001 | 0.86 (0.81–0.91) | <0.001 |
| Maternal age | 0.88 (0.84–0.92) | <0.001 | — | — |
| Paternal age | 0.90 (0.87–0.93) | <0.001 | — | — |
| Sibling count (per additional sibling) | 0.77 (0.67–0.88) | <0.001 | — | — |
| Sibling count ≥3 | 0.44 (0.29–0.67) | <0.001 | 0.60 (0.38–0.94) | 0.027 |
| Incident location: school vs home | 0.45 (0.25–0.83) | 0.011 | 0.91 (0.46–1.80) | 0.781 |
| Incident location: outdoors vs home | 0.56 (0.35–0.88) | 0.012 | 0.79 (0.48–1.31) | 0.368 |

OR: Odds ratio; CI: Confidence interval; VIF: Variance inflation factor. Adjusted OR values were calculated using a multivariable logistic regression model.

Prior to multivariable modelling, correlations among age-related variables (child age, maternal age, paternal age, family age difference, and family age mean) were examined using Pearson and Spearman correlation coefficients. Maternal and paternal age were highly correlated (Pearson’s r = 0.89, p < 0.001) and strongly correlated with the composite family age mean variable (r = 0.97–0.98, p < 0.001). Both maternal and paternal age also demonstrated moderate correlations with child age (r ≈ 0.58–0.61, p < 0.001). To avoid multicollinearity, maternal and paternal age were excluded from the multivariable model and child age was retained as the primary age-related predictor.

When paternal employment and family employment status were simultaneously included in the model, extremely high variance inflation factors (VIF ≈ 939) and very low tolerance values (~0.001) indicated near‑perfect multicollinearity. Because these variables represent closely related indicators of household socioeconomic status, only paternal employment was retained in the final multivariable model. After removal of family employment status, VIF values for the remaining predictors ranged between approximately 1.00 and 1.11, indicating no evidence of problematic multicollinearity.

A multivariable logistic regression model was constructed to identify factors associated with head injury. The overall model was statistically significant (χ²(6) = 56.93, p < 0.001). Model performance showed modest explanatory power (McFadden R² = 0.11; Cox–Snell R² = 0.13; Tjur R² = 0.13) with acceptable discrimination (AUC = 0.72). The Akaike Information Criterion (AIC) was 485.64.

# Supplementary Table S3. Baseline characteristics according to specialist consultation requirement in the emergency department (N = 408)

| Variable | Category | No consultation (n=364) | Consultation required (n=44) | Total | p value | | Effect size |
| --- | --- | --- | --- | --- | --- | --- | --- |
| Age (years) | Median (IQR) | 8.0 (4.0–11.0) | 9.0 (4.5–11.0) | 8.0 (4.0–11.0) | | 0.897 | 0.01 |
| Sex | Female | 141 (38.7) | 13 (29.5) | 154 (37.7) | | 0.306 | 0.06 |
|  | Male | 223 (61.3) | 31 (70.5) | 254 (62.3) | |  |  |
| Incident location | Home | 173 (47.5) | 21 (47.7) | 194 (47.5) | | 0.747 | 0.04 |
|  | School | 65 (17.9) | 6 (13.6) | 71 (17.4) | |  |  |
|  | Outdoors | 126 (34.6) | 17 (38.6) | 143 (35.0) | |  |  |
| Trauma mechanism | Fall | 264 (72.5) | 38 (86.4) | 302 (74.0) | | 0.260 | 0.10 |
|  | Impact | 97 (26.6) | 6 (13.6) | 103 (25.2) | |  |  |
|  | Motor vehicle accident | 2 (0.5) | 0 (0.0) | 2 (0.5) | |  |  |
|  | Bicycle injury | 1 (0.3) | 0 (0.0) | 1 (0.2) | |  |  |
| Sibling count | Median (IQR) | 2.0 (1.0–3.0) | 3.0 (2.0–4.0) | 2.0 (2.0–3.0) | | 0.007 | 0.24 |
| Sibling ≥3 | No | 204 (56.0) | 14 (31.8) | 218 (53.4) | | 0.004 | 0.15 |
|  | Yes | 160 (44.0) | 30 (68.2) | 190 (46.6) | |  |  |
| Maternal age (years) | Median (IQR) | 35.0 (30.0–40.0) | 36.5 (33.8–40.0) | 35.0 (30.0–40.0) | | 0.177 | 0.12 |
| Paternal age (years) | Median (IQR) | 39.0 (34.0–44.0) | 40.5 (36.8–45.0) | 39.0 (35.0–44.2) | | 0.106 | 0.15 |
| Maternal employment | Unemployed | 318 (87.4) | 40 (90.9) | 358 (87.7) | | 0.664 | 0.03 |
|  | Employed | 46 (12.6) | 4 (9.1) | 50 (12.3) | |  |  |
| Paternal employment | Unemployed | 19 (5.2) | 4 (9.1) | 23 (5.6) | | 0.480 | 0.05 |
|  | Employed | 345 (94.8) | 40 (90.9) | 385 (94.4) | |  |  |
| Family employment | Unemployed | 17 (4.7) | 4 (9.1) | 21 (5.1) | | 0.372 | 0.06 |
|  | Employed | 347 (95.3) | 40 (90.9) | 387 (94.9) | |  |  |
| Caregiver availability | No caregiver | 343 (94.2) | 42 (95.5) | 385 (94.4) | | 1.000 | 0.02 |
|  | Has caregiver | 21 (5.8) | 2 (4.5) | 23 (5.6) | |  |  |

Continuous variables are presented as median with interquartile range (25th–75th percentiles), and categorical variables are presented as number (%). Group comparisons were performed using the Mann–Whitney U test for continuous variables and chi-square tests for categorical variables. Effect sizes are reported as rank biserial correlations for Mann–Whitney U tests and Cramér’s V for chi-square tests.

# Supplementary Table S4. Multivariable logistic regression analysis of factors associated with specialist consultation requirement

| Variable | Univariate OR (95% CI) | P value | Adjusted OR (95% CI) | P value |
| --- | --- | --- | --- | --- |
| Male sex | 1.51 (0.76–2.98) | 0.273 | 1.67 (0.83–3.37) | 0.149 |
| Age | 0.99 (0.93–1.07) | 0.963 | 0.96 (0.88–1.03) | 0.260 |
| Sibling count (per additional sibling) | 1.30 (1.08–1.56) | 0.006 | 1.35 (1.11–1.64) | 0.003 |
| Sibling count ≥3 | 2.73 (1.40–5.32) | 0.003 | 3.03 (1.51–6.07) | 0.002 |

OR: Odds ratio; CI: confidence interval. Adjusted OR values were obtained using multivariable logistic regression analysis including age, sex, and sibling count variables.
